# Molecular Landscape and Predictive Significance of Programmed Cell Death‐Related Genes in Sepsis

**DOI:** 10.1155/humu/5280021

**Published:** 2026-01-13

**Authors:** Shiqiang Min, Tao Zhang, Song Chen

**Affiliations:** ^1^ Department of Emergency and Critical Care Medicine, Shanghai Pudong New Area People′s Hospital, Shanghai, China

**Keywords:** immune cell infiltration, machine learning, programmed cell death, sepsis, single-cell RNA sequencing

## Abstract

Sepsis, a systemic inflammatory response to infection, remains a significant health challenge with high morbidity and mortality rates. The molecular mechanisms underlying sepsis, particularly the role of programmed cell death (PCD), are not fully understood. This study is aimed at elucidating the transcriptomic changes associated with sepsis, emphasizing PCD, and identifying potential diagnostic biomarkers. Transcriptome data from sepsis and control samples were extracted from the GEO website. Differential expression analysis identified genes perturbed in sepsis. WGCNA revealed 14 highly connected modules, with the turquoise module showing the strongest association with sepsis. A set of 262 hub genes was identified, which were mainly associated with apoptotic signaling pathways. Seven prognostic‐related overlapping feature genes (PRGs) were identified. More importantly, the diagnostic model, constructed using eight machine learning algorithms, exhibited high efficacy in distinguishing sepsis patients from controls. The validation of feature genes at the scRNA‐seq level adds a layer of robustness to our conclusions. The strong association of genes like S100A9 and KLHL3 with neutrophils, pivotal players in sepsis, suggests potential avenues for therapeutic targeting. Our comprehensive analysis has unveiled the significant role of PCD in sepsis. The insights gained from this study provide a foundation for future therapeutic interventions.

## 1. Introduction

Sepsis, a life‐threatening condition resulting from the overwhelming response to an infection, stands as a significant global health challenge [1]. Characterized by a systemic inflammatory response, sepsis can lead to multiple organ dysfunction and, if not promptly addressed, can result in high morbidity and mortality rates [2, 3]. Despite advancements in medical science, the molecular mechanisms underlying sepsis remain incompletely understood, making its early diagnosis and effective treatment challenging [4, 5].

Programmed cell death (PCD) is a fundamental biological process that plays a pivotal role in maintaining cellular homeostasis by eliminating damaged or unnecessary cells. There are several forms of PCD, including apoptosis, pyroptosis, ferroptosis, autophagy, necroptosis, cuproptosis, parthanatos, entotic cell death, NETotic cell death, lysosome‐dependent cell death, alkaliptosis, oxeiptosis, and disulfidptosis, each with distinct molecular pathways and physiological roles [6, 7]. Ferroptosis, inspired by iron‐mediated phospholipid peroxidation, is governed by diverse metabolic signaling pathways encompassing oxidative balance, iron management, mitochondrial functions, and the metabolism of amino acids, fats, and carbohydrates. Moreover, it is intertwined with various signaling pathways pertinent to illnesses. A myriad of tissue damages and degenerative conditions are propelled by this process [8]. Cuproptosis is implemented by damaged copper hematemesis where surplus intracellular copper encourages the clumping of lipoylated dihydrolipoamide S‐acetyltransferase (DLAT), a protein linked to the mitochondrial tricarboxylic acid (TCA) cycle and proteotoxic distress [9]. Parthanatos is a unique cell death mechanism that relies on PARP1 and operates independently of caspases, setting it apart from apoptosis, necrosis, and other recognized cell death processes, where several molecules such as PARP1, ARH3, AIF, and MIF are involved [10]. Entotic cell death is defined as a novel cell removal mechanism where breast cancer cells infiltrate adjacent cells by creating cell‐in‐cell structures. This process is mediated by the epithelial adherens junction (AJ), which comprises epithelial‐cadherin (E‐cadherin) and the AJ/cytoskeleton‐associated protein *α*‐catenin [11]. NETotic cell death is induced by the rapid formation of neutrophil extracellular traps (NETs), which is caused by the release of nuclear and mitochondrial DNA adorned with granule and cytosolic proteins in neutrophils [12]. Lysosome‐driven cell death is facilitated by lysosomal content that is released in the cytosol when the lysosome membrane becomes permeable [13]. The autophagy process involves a complex lysosomal degradation process, aiding in metabolic adjustments and the recycling of nutrients. Alkaliptosis is influenced by pH and activated by the compound JTC801 [14]. There are no commonly recognized cell death effectors in alkaliptosis, which is primarily driven by two key signaling pathways: the NF‐*κ*B‐CA9 and the ATP6V0D1‐STAT3 pathways [15]. Oxeiptosis is a cell death pathway that is responsive to reactive oxygen species and plays a crucial role in defending against inflammation. It operates independently of caspases and does not trigger the inflammation response [16]. Disulfidptosis is characterized by the disulfide stress and a rise in disulfide bonds within the actin cytoskeleton under glucose deprivation [17].

Dysregulation of PCD can lead to various pathological conditions, including cancer, neurodegenerative diseases, and immune disorders. In the context of sepsis, aberrant PCD can contribute to the excessive loss of immune cells, leading to immunosuppression, or can result in the premature death of essential tissue cells, exacerbating organ dysfunction. Yet, a thorough overview of the connection between PCD and sepsis is still elusive, and the specific roles of PCD in sepsis have not been extensively explored. In this study, we embarked on a comprehensive transcriptomic analysis of sepsis, aiming to identify the hub PCD genes associated with the sepsis prognosis and diagnosis, with a particular emphasis on potential candidates of biomarkers. In summary, our study depicts the heterogeneity across sepsis and validates the clinical prognosis of PCD genes. Further, we sought to elucidate the molecular underpinnings of sepsis and identify potential therapeutic targets.

## 2. Materials and Methods

### 2.1. Sample Collection And Data Processing

The transcriptome data of the sepsis sample and corresponding control groups were extracted from the GEO dataset. To guarantee a rigorous analysis, we selected GSE57065 and GSE95233 for their substantial sample sizes and extensive clinical data. Specifically, GSE57065, employed as the training dataset, comprises 107 samples, including 25 healthy controls and 82 sepsis patients. GSE95233, used for validation, contains 124 samples, with 22 controls and 102 sepsis patients. In addition, we validated the hub gene expression in the single‐cell sequencing dataset GSE167363. Genes of 12 PCD ways were acquired from a previous study [18]. In addition, disulfidptosis‐associated genes were obtained from a recent report [7]. The programmed cell death–related genes (PCDGs) are also shown in Table S1.

### 2.2. Differential Expression Analysis and Weighted Gene Coexpression Network Analysis (WGCNA) Analysis

Differentially expressed genes (DEGs) between sepsis (named as P) and control (named as C) group in GSE57065 were identified according to the following criteria: logFC > 0.5 & adj. *p* < 0.05. Then, we performed gene set enrichment analysis (GSEA) of the DEGs to explore the predominant signaling pathways. Furthermore, the GSEA of the up or down DEGs was also visualized. WGCNA was used to construct a weighted gene coexpression network and screened out the key gene module. The soft thresholding power was determined to be 7, optimizing the scale‐free *R* ^2^ criterion to approach 0.90. Modules were identified using a dynamic tree‐cutting algorithm, with the minimum module size established at 50 genes to ensure the detection of biologically meaningful modules. Module–trait relationships were assessed using gene significance and module membership measures.

### 2.3. Hub Gene Identification and Functional Analysis

Hub genes, central to the network, were identified as the intersection of DEGs (|logFC| >0.5, adj. *p* < 0.05), PCDGs (Table S1), and genes from the turquoise module in WGCNA (module internal connectivity kME > 0.8). The STRING database was utilized to construct PPI networks, visualizing interaction degrees among the hub genes. Gene ontology (GO) and KEGG pathway enrichment analyses were performed to elucidate the biological roles of these hub genes. Disease ontology (DO) analysis was used to investigate the major diseases these hub genes involved [19].

### 2.4. Machine Learning Methods Identified Feature Genes

Multiple machine learning algorithms were utilized to identify feature genes associated with sepsis prognosis. Lasso regression (10‐fold cross‐validation with the 1‐SE rule *λ* selection criterion), known for its variable selection and regularization capabilities, uses cross‐validation to optimize the lambda parameter, selectively retaining genes with nonzero coefficients to prevent model overfitting. The SVM‐RFE (linear kernel, recursive feature elimination until accuracy drops by over 5%) algorithm refines the feature set by iteratively removing the least significant features, thereby enhancing the model′s predictive accuracy. This approach was pivotal in isolating the most informative genes for distinguishing sepsis from controls. Random forest (RF) (ntree = 500, mtry = √*p*, using MeanDecreaseGini > 0.1 as the threshold for feature importance), an ensemble learning method, effectively handles unbalanced datasets and provides estimates of feature importance, crucial for pinpointing key genes. The Boruta algorithm (iterations = 100, maximum importance shadow area), dedicated to robust feature selection, identifies genes that significantly differentiate sepsis from controls. Genes consistently selected across these four methods were designated as feature genes for further analysis.

### 2.5. Expression Validation of Feature Genes

The expression profiles of the identified feature genes were validated in two independent datasets (GSE57065 and GSE95233). Also, we verified their expression abundance at the scRNA level (GSE167363). The diagnostic significance of these genes was assessed by plotting ROC curves, and the area under the curve (AUC) values was calculated. The correlation heatmap between feature genes in the sepsis sample was also visualized.

### 2.6. Prognostic Model Construction

To generate an optimal diagnostic model with the best efficacy, we introduced eight machine learning algorithms such as Kknn, Ida, SVM, XGboost, ranger, and naïve Bayes to screen the appropriate algorithm that obtains the highest diagnostic efficacy [20]. The diagnostic significance of the model was assessed by plotting ROC curves, and the AUC value of the model was calculated.

### 2.7. Immune Cell Infiltration Analysis

The CIBERSORT algorithm was employed to estimate the proportions of 22 immune cell types in the sepsis and control samples. The relative abundance of each cell type was compared between the two groups. Additionally, we analyzed the association between feature genes and immune cell types in the sepsis sample.

### 2.8. Identification of Hallmark Gene Sets

To explore the enrichment differences in hallmark gene sets between sepsis and control groups, ssGSEA was employed. The method calculates separate enrichment scores for each pairing of a sample and gene set, allowing for the identification of pathways that are coordinately upregulated or downregulated within each sample. The Molecular Signatures Database (MSigDB) was utilized, specifically focusing on the collection of 50 hallmark gene sets that summarize and represent specific well‐defined biological states or processes. To understand the relationship between the identified feature genes and the hallmark gene sets, a correlation analysis was performed. Spearman′s rank correlation was used to determine the association between the expression of each feature gene and the enrichment score of each hallmark gene set.

### 2.9. ssGSEA Analysis of Feature Genes

Given the notable findings related to feature genes, sepsis samples were divided into two groups based on the median expression value of each feature gene. The DEGs between the high and low expression groups were obtained to perform GSEA analysis.

### 2.10. Single‐Cell RNA Sequencing (scRNA‐Seq) Analysis

The raw scRNA‐seq data from the GSE167363 dataset were processed using the Seurat package in R. We evaluated key metrics, such as nCount RNA and nFeature RNA, and assessed mitochondrial contamination by calculating the percentage of reads mapping to mitochondrial genes. This step was crucial for identifying and filtering low‐quality or dead cells, thus ensuring data integrity (for each sample, cells with 200–5000 retained genes were kept and cells with mitochondrial proportion > 20% or red blood cell proportion > 3% were excluded). After rigorous quality control and normalization, batch effects were corrected using the Harmony package. For cell clustering, dimensionality reduction via t‐SNE was performed after principal component filtering, enabling precise visual delineation of cell clusters. Statistically significant cell marker genes with strong discriminatory power were identified using the FindAllMarkers function in Seurat, employing a nonparametric Wilcoxon rank sum test with Bonferroni correction. The composition of cell types within each cluster was quantitatively displayed. Additionally, we analyzed the expression of feature genes across various cell types, revealing distinct expression patterns. A *p* value <0.05 was considered statistically significant.

### 2.11. The Expression of Key Genes Was Verified by qPCR

A total of 10 patients with sepsis and 10 without sepsis were enrolled from the intensive care unit (ICU). Exclusion criteria included age < 18 years, ICU stay < 24 h, pregnancy/lactation, malignancy, immunosuppressive therapy, or HIV infection.

Total RNA was extracted from whole blood samples of enrolled patients using RNAiso Plus reagent (TaKaRa, Tokyo, Japan), strictly following the manufacturer′s protocol. Subsequently, cDNA was synthesized using a reverse transcription kit (US Everbright, Suzhou, China). Quantitative PCR was performed with the Universal SYBR Green qPCR SuperMix kit (US Everbright, Suzhou, China), adhering to the kit′s operating guidelines. RT‐qPCR analysis was performed on the Applied Biosystems QuantStudio 3 system (Thermo Fisher Scientific, United States). Relative gene expression levels were calculated using the 2^−*Δ* *Δ*Ct^ method. Primer sequences are detailed in Table S2.

### 2.12. Statistical Analysis

Statistical analysis of experimental data was performed using GraphPad Prism software (Version 10). Public datasets were processed and visualized using R software (Version 4.4.0). Statistical significance in the figures is denoted as *p* < 0.05 (∗), *p* < 0.01 (∗∗), and *p* < 0.001 (∗∗∗).

## 3. Results

### 3.1. GSEA of DEGs Between Sepsis and Control Group

By performing differential expression analysis, a total of 3046 DEGs were identified. A total of 1389 DEGs such as NOV, SEPT9, XIST, ESYT1 were upregulated in sepsis, whereas 1657 DEGs such as S100A9, IRAK3, MCEMP1, TLR5, CD177 were downregulated (Figures 1a,b). GSEA investigation suggested that these DEGs were mainly associated with fatty acid biosynthesis; graft‐versus‐host disease; intestinal immune network for IgA production; legionellosis; mucin‐type O‐glycan biosynthesis; nitrogen metabolism; pantothenate and CoA biosynthesis; PPAR signaling pathway; primary immunodeficiency; starch and sucrose metabolism; Th1, Th2, and Th17 cell differentiation; Type I diabetes mellitus; ubiquinone and other terpenoid‐quinone biosynthesis; and viral myocarditis (Figure 1c). The significant KEGG pathways were also displayed such as complement and coagulation, cascades, starch and sucrose metabolism, legionellosis, PPAR signaling pathway, NET formation, pantothenate and CoA biosynthesis, fluid shear stress, and atherosclerosis (Figure 1d). The upregulated DEGs were associated with antigen processing and presentation; autoimmune thyroid disease; graft‐versus‐host disease; an intestinal immune network for IgA production; primary immunodeficiency; Th1, Th2, and Th17 cell differentiation; Type I diabetes mellitus; and viral myocarditis pathways (Figure 1e). The downregulated DEGs were principally related to complement and coagulation cascades, fatty acid biosynthesis, legionellosis, mucin‐type O‐glycan biosynthesis, nitrogen metabolism, pantothenate and CoA biosynthesis, PPAR signaling pathway, starch and sucrose metabolism, ubiquinone, and other terpenoid‐quinone biosynthesis (Figure 1f). Detailed results of the GSEA analysis can be found in Table S3.

Figure 1The GESA results of DEGs between sepsis and control group. (a) Heatmap of DEGs, (b) volcano plot of DEGs, (c) KEGG results of DEGs, (d) ridgeline plot of GSEA, and (e,f) KEGG results of upregulated and downregulated DEGs, respectively.

**Figure:**
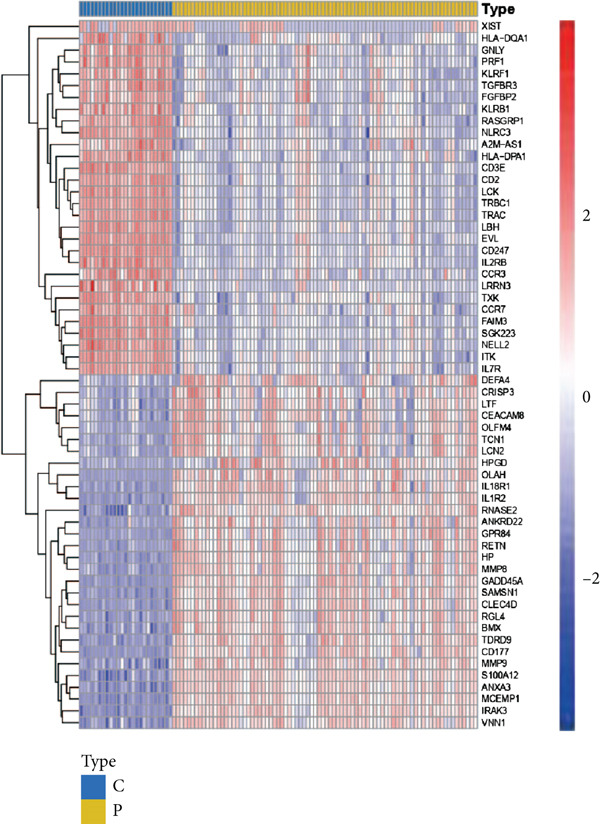
(a)

**Figure:**
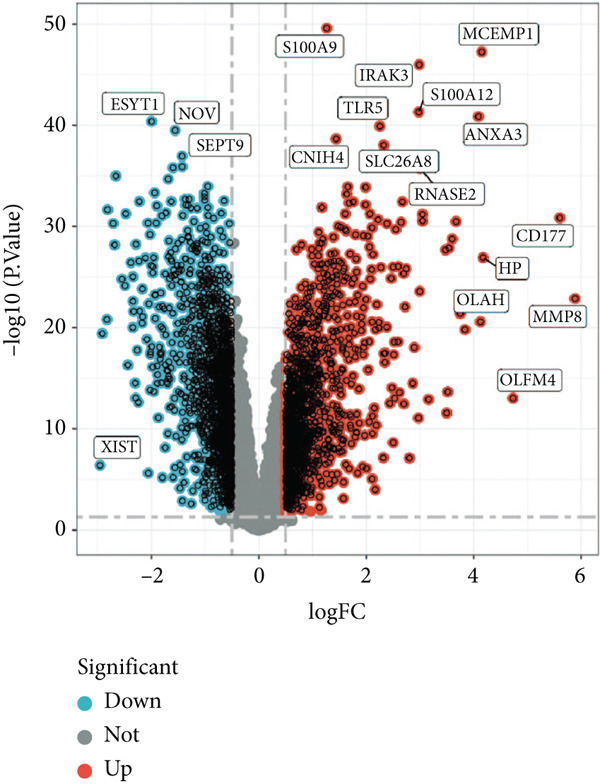
(b)

**Figure:**
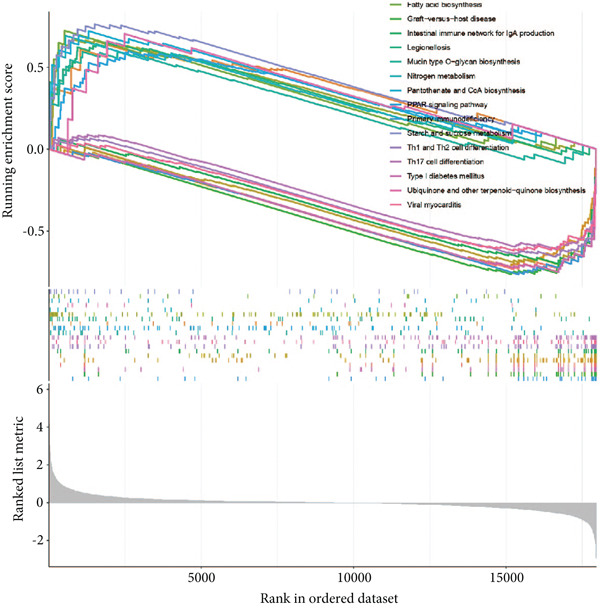
(c)

**Figure:**
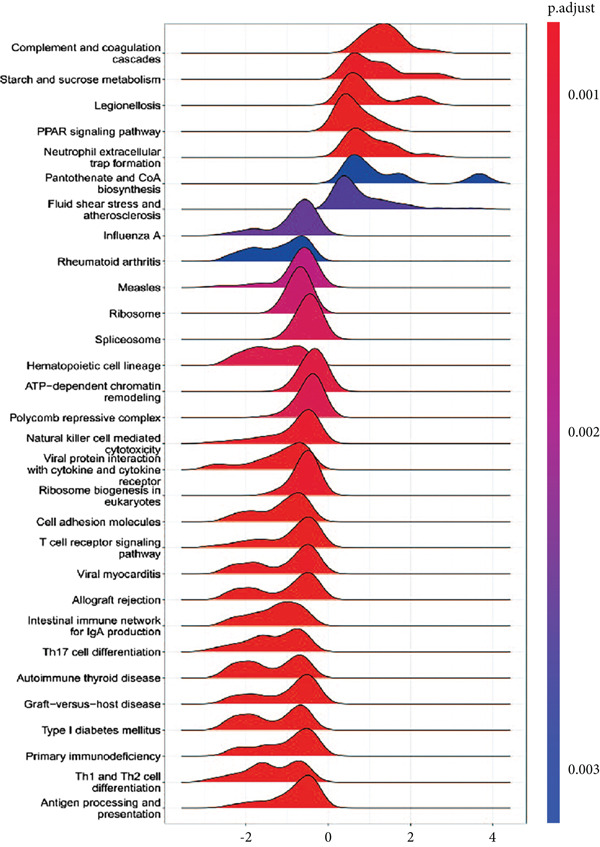
(d)

**Figure:**
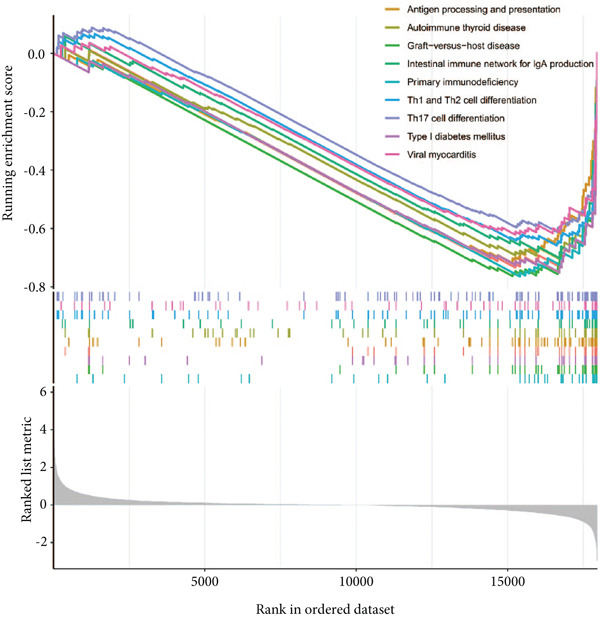
(e)

**Figure:**
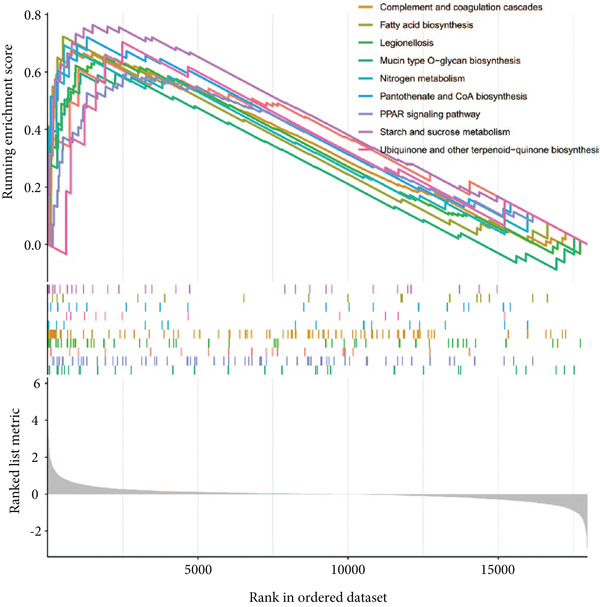
(f)

### 3.2. Establishment of Key Gene Module by WGCNA

We conducted WGCNA to establish the weight correlation work of DEGs. The DEG patterns could well distinguish the samples into sepsis and control group (Figure 2a). Then, according to the scale‐free *R* ^2^ = 0.9, the soft power value was established as 7 (Figure 2b), and minModuleSize = 50, 14 highly connectivity modules were identified for the following analysis (Figure 2c). The correlation analysis validated the independence of each gene module (Figure 2d). The correlation analysis of module feature genes validated the reliability of the modules definition (Figure 2e). Among 14 modules, the ME turquoise module displayed the strongest association with sepsis (Figure 2f,g). The genes of the turquoise module were used in the subsequent analysis.

Figure 2WGCNA identified the key gene modules. (a) Sample clustering dendrogram with clinical traits, (b) establishment of the scale‐free fit index and the mean connectivity, (c) cluster dendrogram of gene modules with minModuleSize = 50, (d) correlation heatmap of transcription analysis of modules, (e) correlation network of module feature genes, (f) heatmap of module‐trait correlations, and (g) gene significance plot of sepsis and control for the turquoise module.

**Figure:**
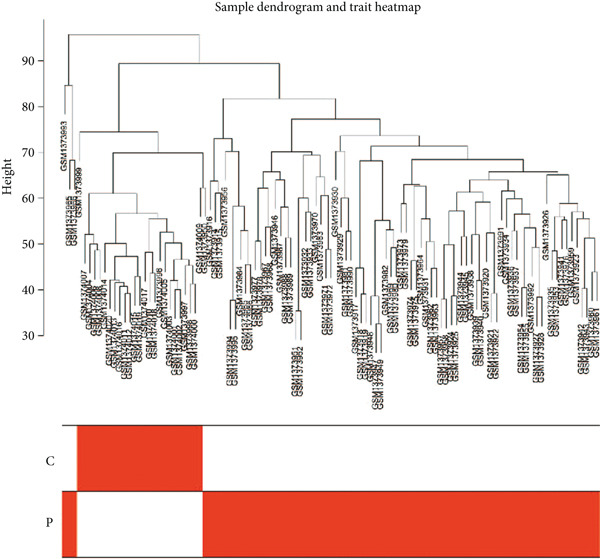
(a)

**Figure:**
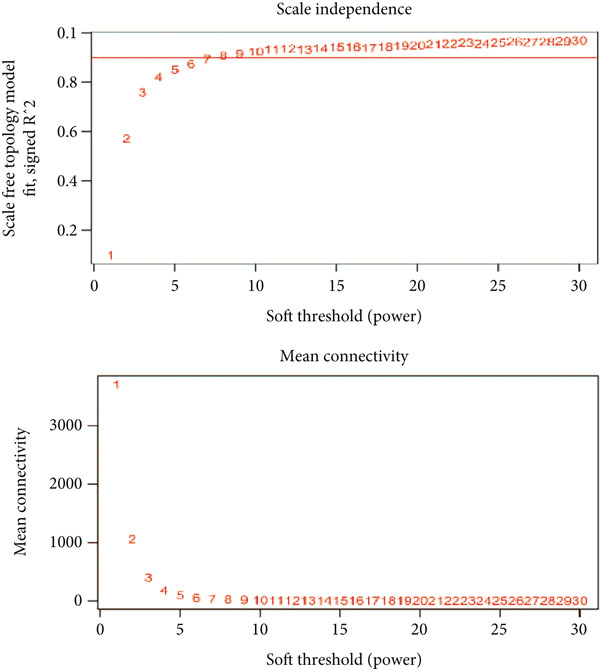
(b)

**Figure:**
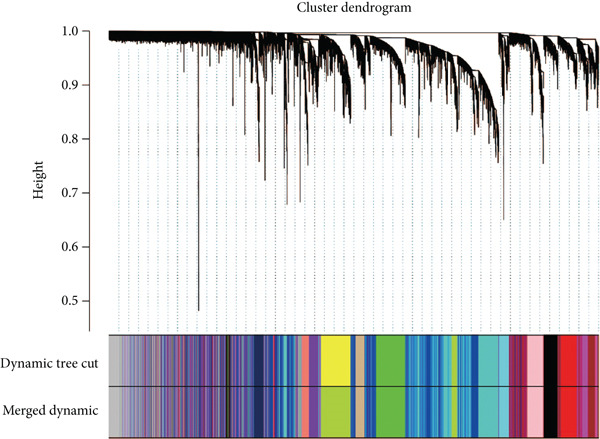
(c)

**Figure:**
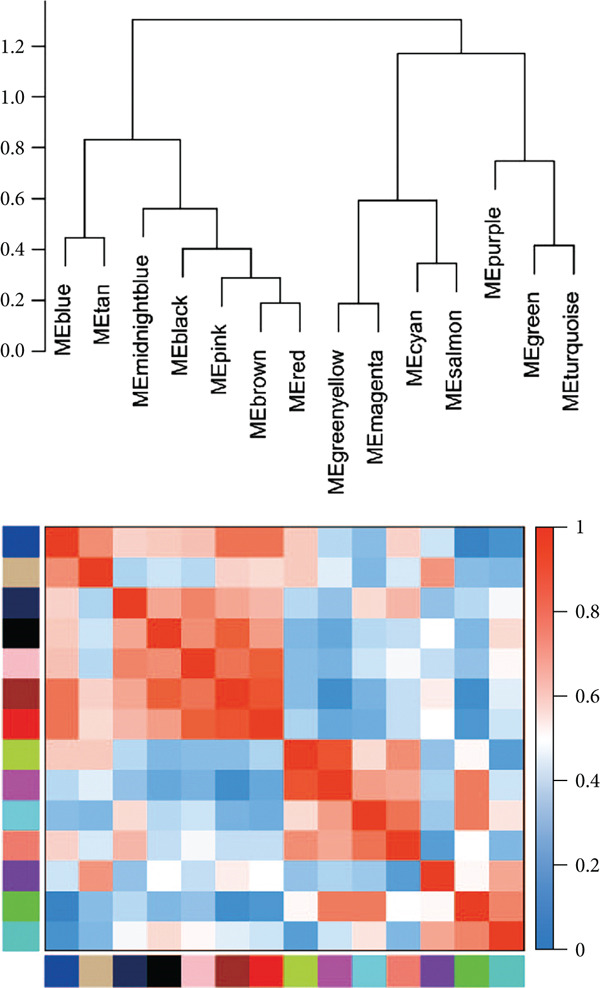
(d)

**Figure:**
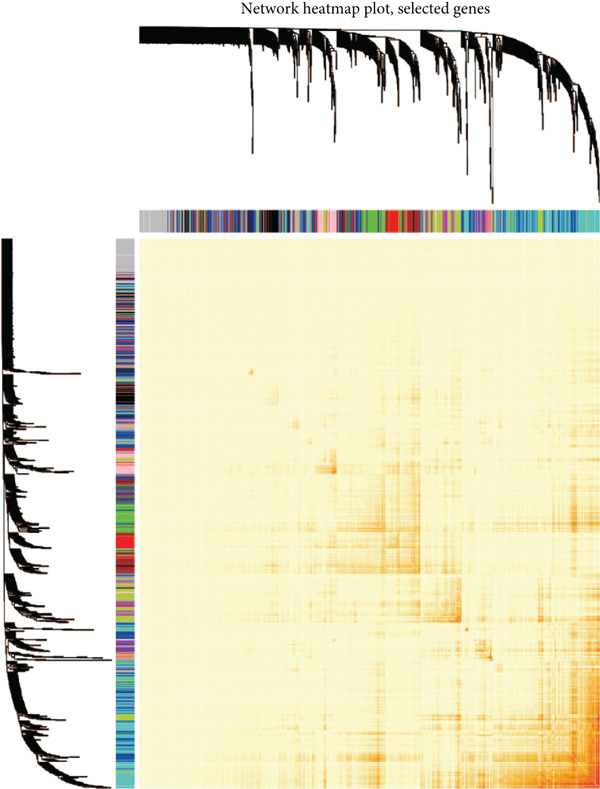
(e)

**Figure:**
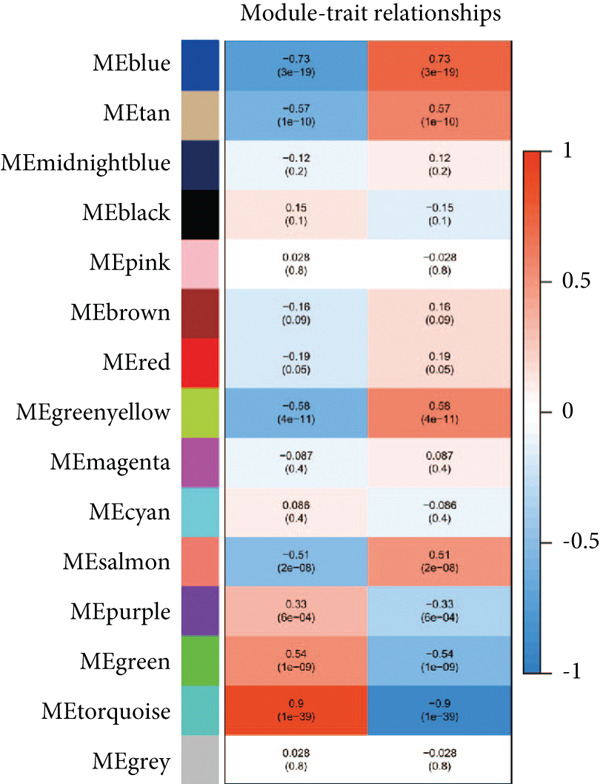
(f)

**Figure:**
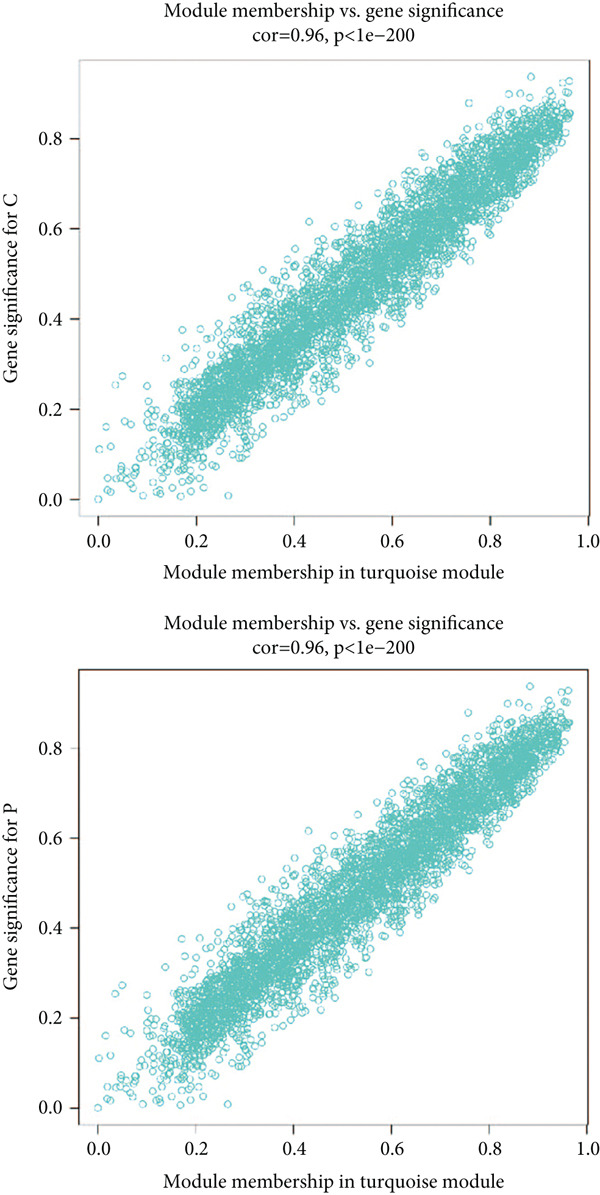
(g)

### 3.3. Enrichment Analysis of Hub Genes

We identified 262 hub genes by taking the intersection between DEGs, PCDGs, and the genes of the turquoise module in WGCNA (Figure 3a). Exhibiting coordinated expression and functional changes in sepsis, these hub genes involved in apoptosis and necrosis, played a crucial role in regulating immune responses and inflammation, particularly within the pathophysiological context of sepsis. A deeper understanding of these features facilitates the elucidation of molecular dynamics underpinning sepsis, thereby identifying promising biomarkers and potential therapeutic targets. Protein–protein interaction analysis showed the correlation intensity of 262 hub genes (Figure 3b,c). Furthermore, GO analysis suggested that these hub genes were mainly associated with the apoptotic signaling pathway for BP, lysosomal membrane, lytic vacuole membrane that engaged in cell death for CC, membrane‐spanning protein tyrosine kinase activity, and ubiquitin‐like protein ligase binding for MF (Figure 3d). On the whole, the hub genes were related to cancer and inflammatory diseases (Figure 3e). KEGG manifested that the hub genes were primarily enriched in apoptosis, necroptosis, human papillomavirus infection, lysosome, hepatitis B, and NOD‐like receptor signaling pathway (Figures 3f,g, and 3h). In addition, the gene expression landscape of hub genes in the significant signaling pathways was also displayed (Figure 3i).

Figure 3Function enrichment of hub genes. (a) Hub genes of DEGs, PCDGs, and genes of the turquoise module; (b) the coexpression network of hub genes; (c) PPI network of hub genes; (d) significant GO terms of hub genes; (e) DO analysis of hub genes; (f–h) KEGG analysis of hub genes; and (i) expression heatmap of KEGG.

**Figure:**
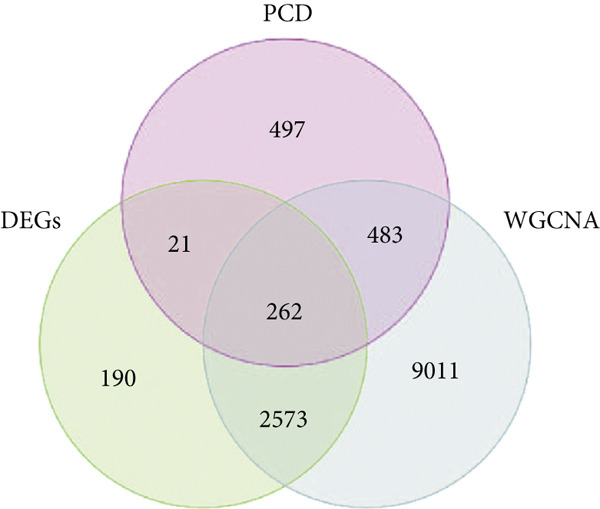
(a)

**Figure:**
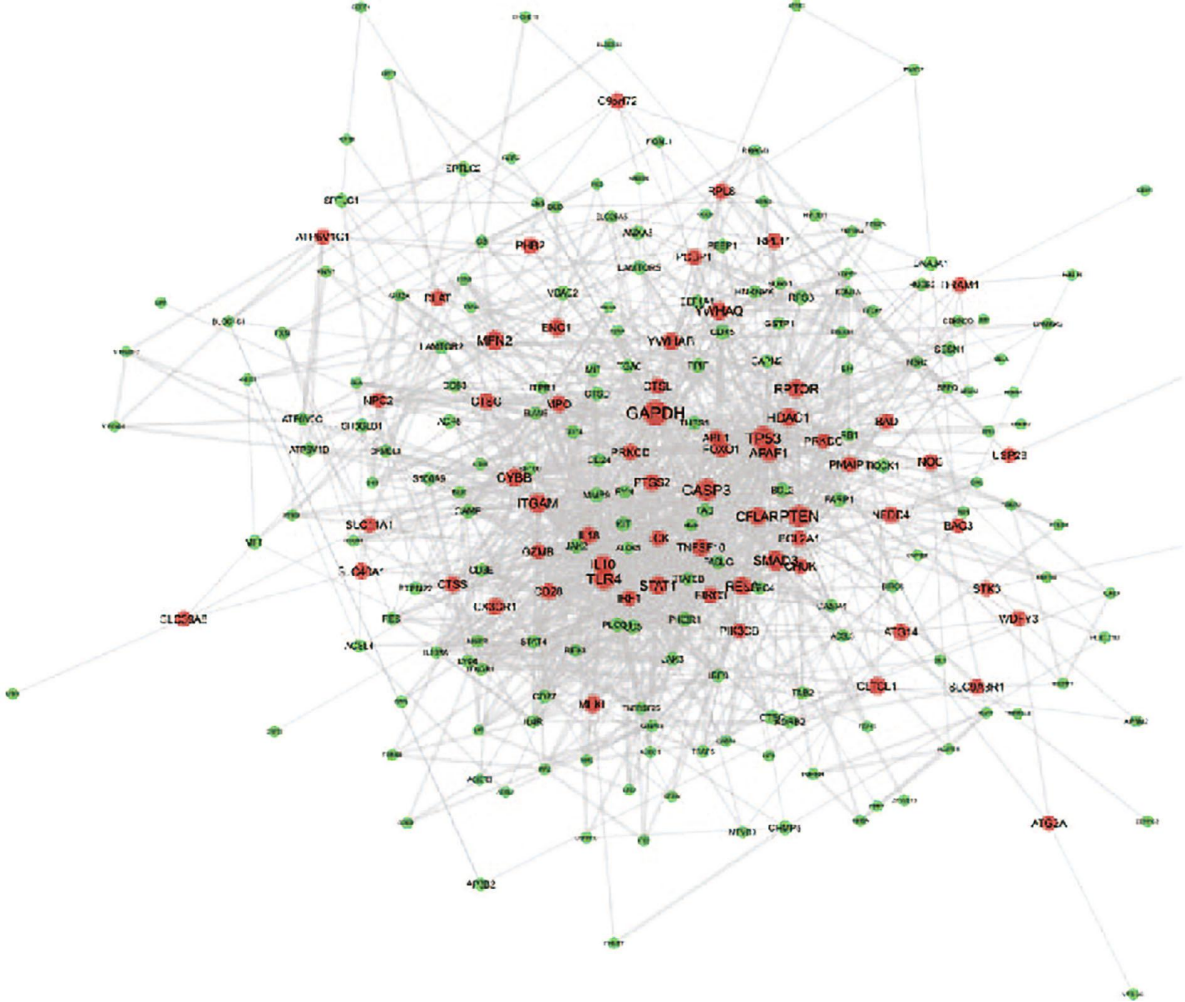
(b)

**Figure:**
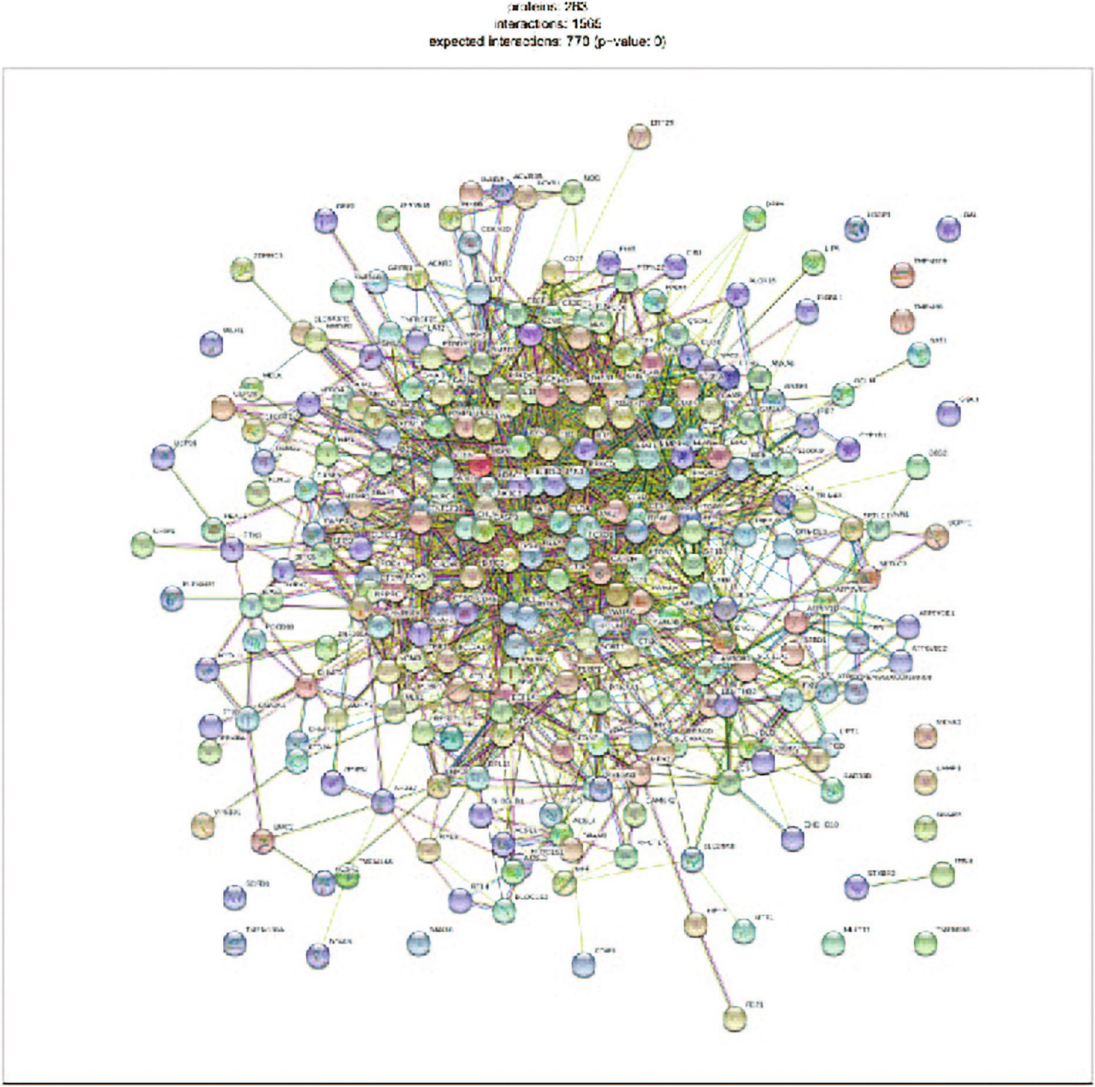
(c)

**Figure:**
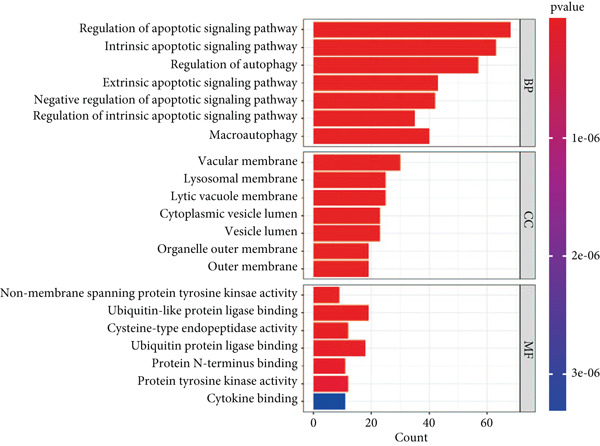
(d)

**Figure:**
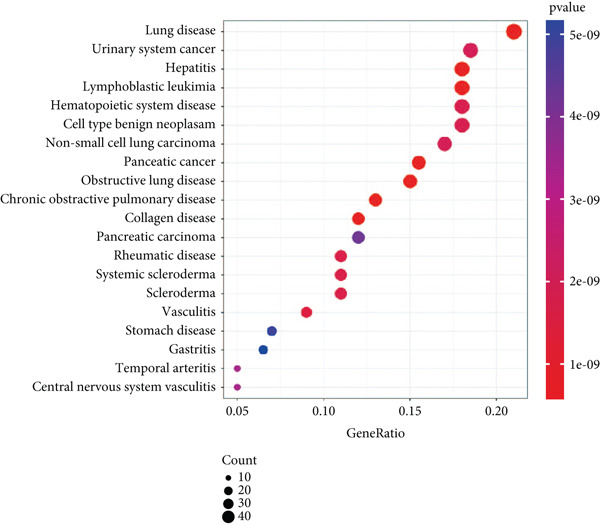
(e)

**Figure:**
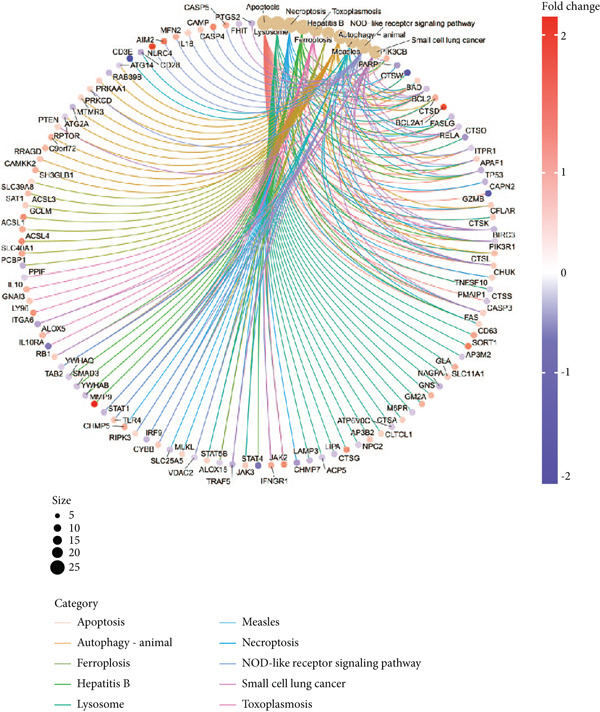
(f)

**Figure:**
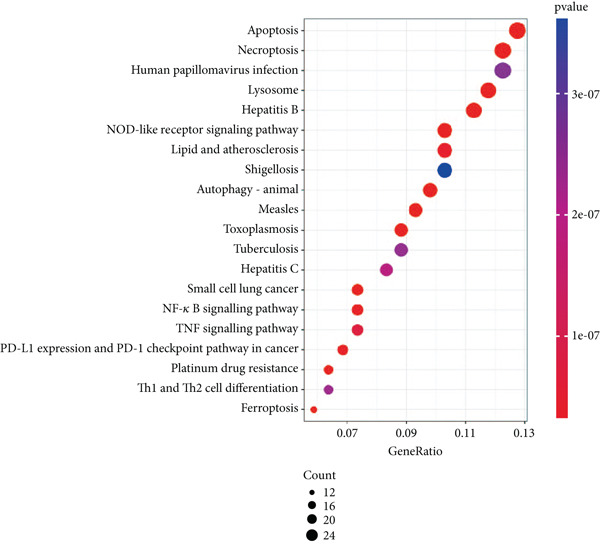
(g)

**Figure:**
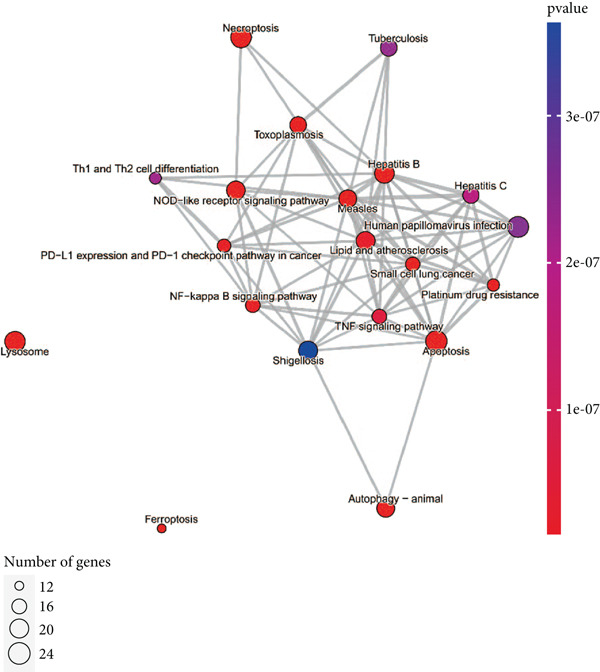
(h)

**Figure:**
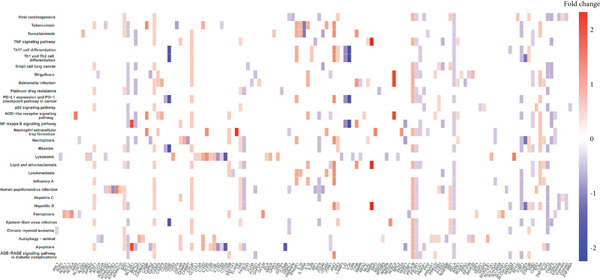
(i)

### 3.4. Feature Gene Identification by Machine Learning Methods

Several machine learning algorithms were employed to screen feature genes among the 262 hub genes. Twelve feature genes were identified by the Lasso approach (Figure 4a). Furthermore, we introduced the SVM‐RFE algorithm; 58 important genes were screened out with the highest accuracy of 0.994 and the lowest error of 0.006 (Figure 4b). Summarily, by implementing RF analysis, we found that 60 genes with importance > 0.1 were related to sepsis survival outcomes (Figure 4c,d). Boruta analysis identified 89 genes that had clinical significance in determining prognosis (Figure 4e). Finally, seven prognostic‐related overlapping feature genes (S100A9, KLHL3, PCBP1, PIK3CB, MTF1, FOXO1, and TMEM59) were identified (Figure 4f,g).

Figure 4Identification of feature genes. (a) Lasso coefficient profiles of the candidate optimal hub genes and the optimal lambda, (b) feature gene selection of SVM algorithm, (c) importance lists of hub genes by RF, (d) random forest for the relationships between the number of trees and error rate, (e) feature gene selection of Boruta algorithm, and (f,g) overlapping feature genes between four machine learning methods.

**Figure:**
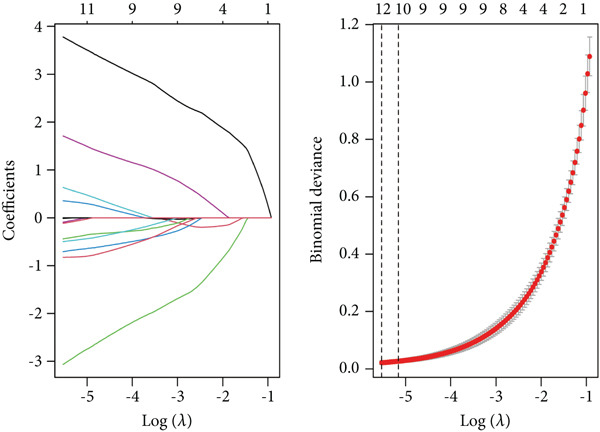
(a)

**Figure:**
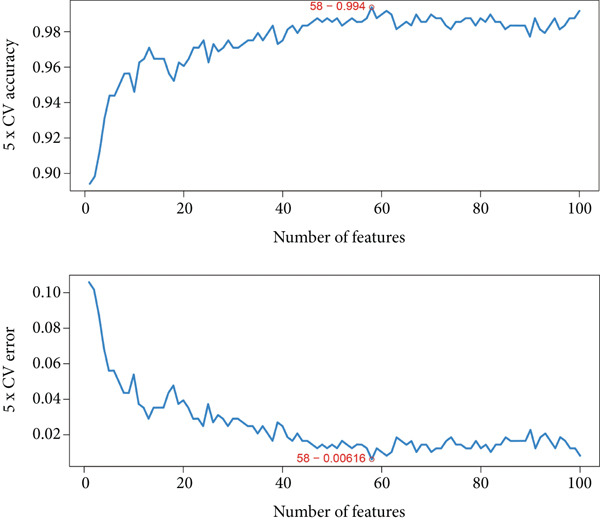
(b)

**Figure:**
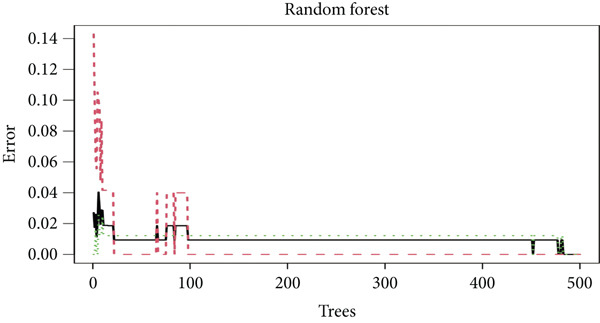
(c)

**Figure:**
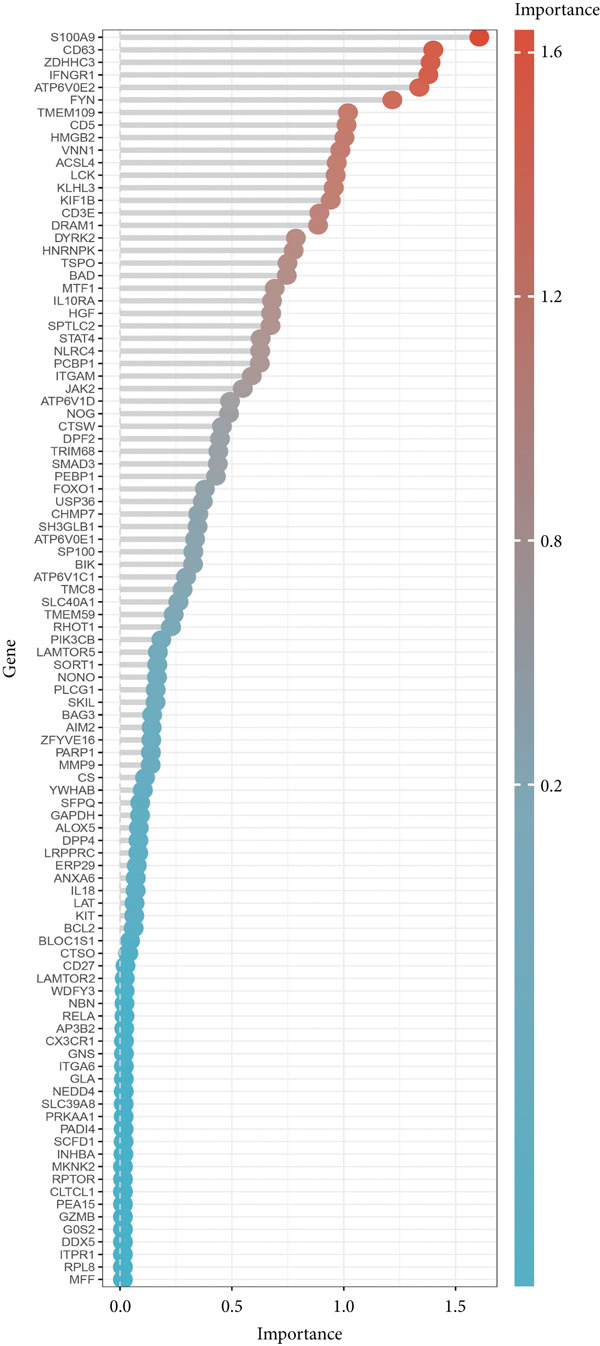
(d)

**Figure:**
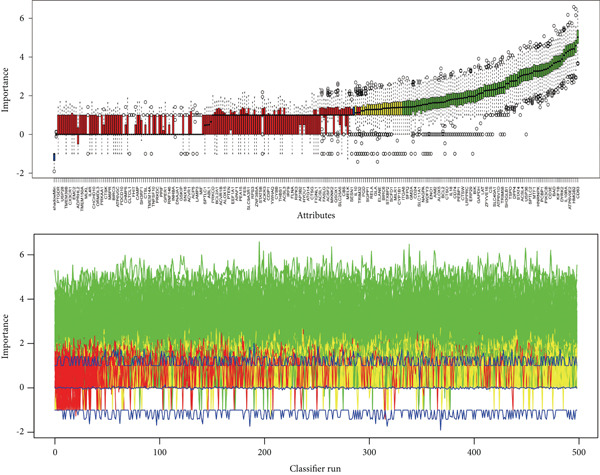
(e)

**Figure:**
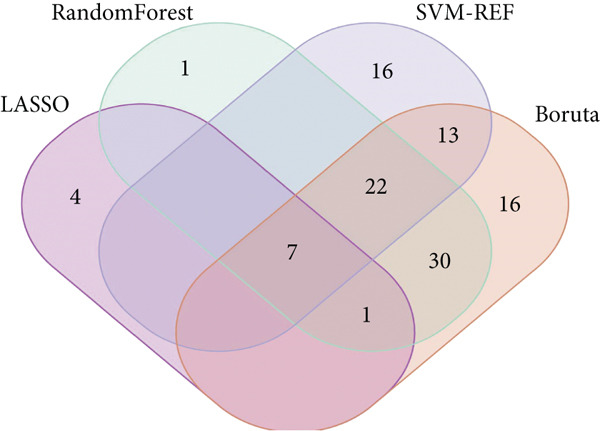
(f)

**Figure:**
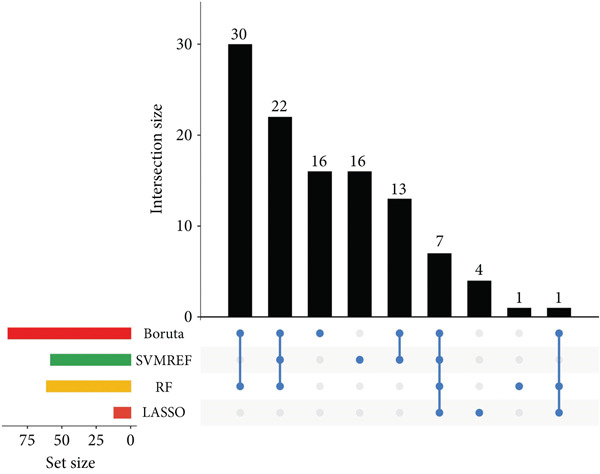
(g)

### 3.5. Validation of Expression Landscape of Seven Feature Genes

To further depict the clinical significance of seven predominant PRGs, we investigated the expression profile of the seven feature genes in both training and validation datasets. FOXO1, KLHL3, and PCBP1 were weakly expressed in the sepsis group whereas MTF1, TMEM59, PIK3CB, and S100A9 were highly expressed in the sepsis group (Figures 5a and 6a). In addition, we tested the diagnostic significance of the seven feature genes. Surprisingly, all of them displayed a high accuracy, evidenced by the more than 0.9 AUC value (Figures 5b and 6b). However, the expression level abundance of the seven feature genes varied across both datasets (Figures 5c and 6c). S100A9 and PCBP1 had the highest transcript abundance whereas KLHL3 had the lowest expression abundance.

Figure 5Expression level of the seven feature genes in GSE57065. (a) Expression bar plot of the seven feature genes, (b) ROC results of the seven feature genes, and (c) expression line plot of the seven feature genes with sample information.

**Figure:**
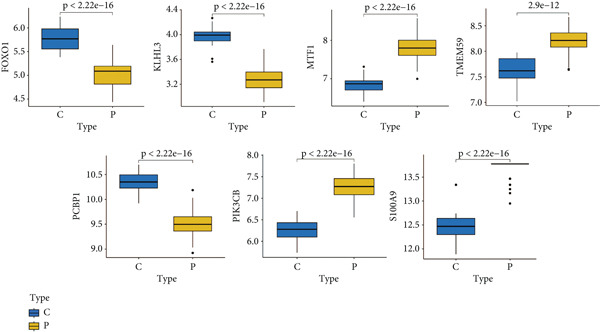
(a)

**Figure:**
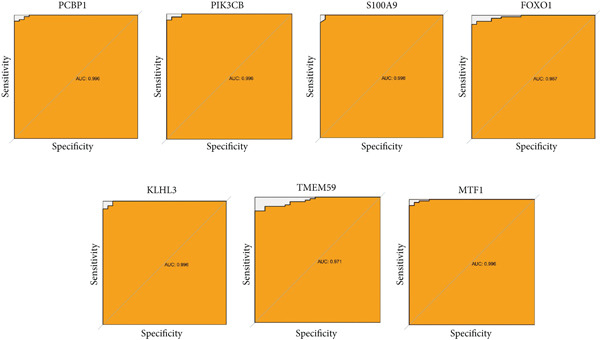
(b)

**Figure:**
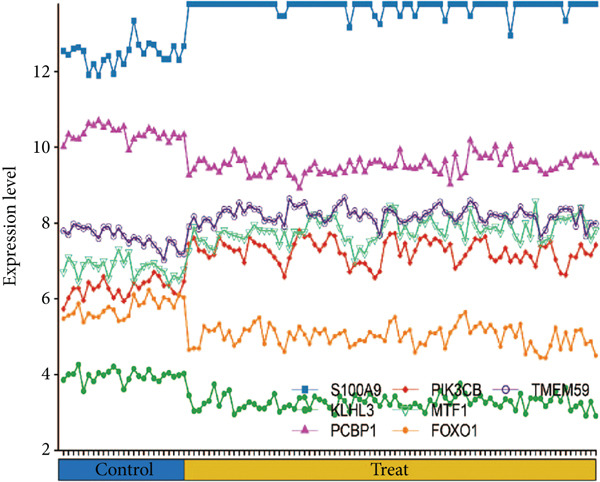
(c)

Figure 6Expression level of the seven feature genes in GSE95233. (a) Expression bar plot of seven feature genes, (b) ROC results of seven feature genes, and (c) expression line plot of feature genes with sample information.

**Figure:**
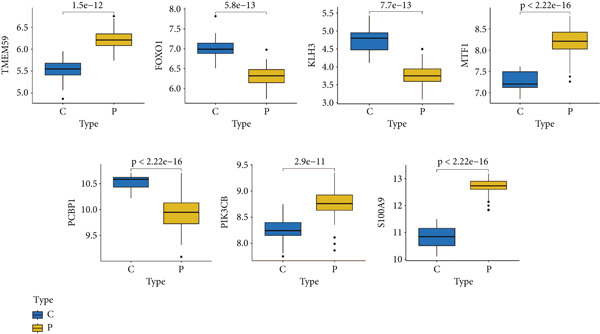
(a)

**Figure:**
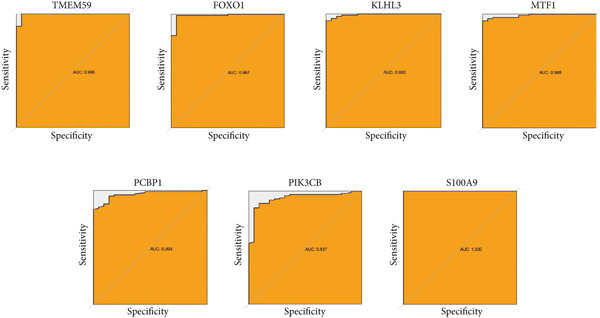
(b)

**Figure:**
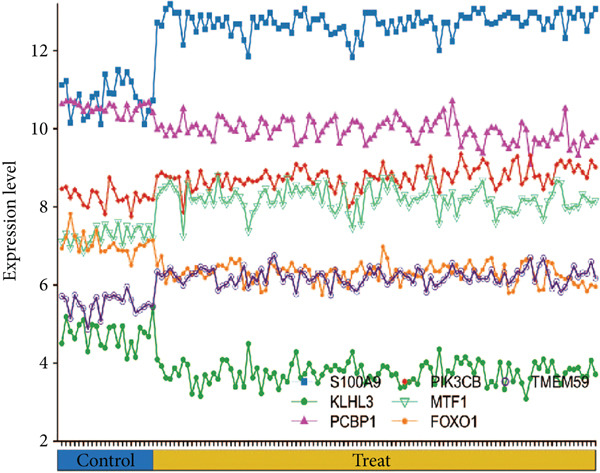
(c)

### 3.6. Identification of Prognostic Model and Immune Cell Infiltration Analysis

Using the seven important feature genes, we developed a prognostic model by applying eight machine learning methods (Figure 7a). The model was trained using dataset GSE57065 and subsequently validated using GSE95233. The prognostic model suggested a powerful diagnostic efficacy and high accuracy (Figure 7b). More importantly, the prognostic signature derived from the seven feature genes could well distinguish the sepsis patients from the control healthy population (Figure 7c). Furthermore, we found that neutrophils were significantly augmented in the sepsis group and had the highest cell amount compared with other types of immune cells (Figure 7d,e), implying the essential role of neutrophils in sepsis progression. Lastly, we surveyed the association between the seven feature genes and immune cell types (Figures 7f and 8a). PIK3B had a significant positive association with macrophages and B cells, whereas a negative association with T cells and NK cells. Also, the interaction of the seven feature genes was explored (Figure 8b).

Figure 7Prognostic model established by seven feature genes and immune cell infiltration analysis. (a) AUC results of eight machine learning methods, (b) ROC analysis of eight machine learning methods, (c) ROC analysis of the prognostic model, (d) common immune cell expression both in sepsis and control groups, (e) the percentages of various types of immune cells in both sepsis and control groups, and (f) correlation heatmap of seven feature genes and various immune cells.

**Figure:**
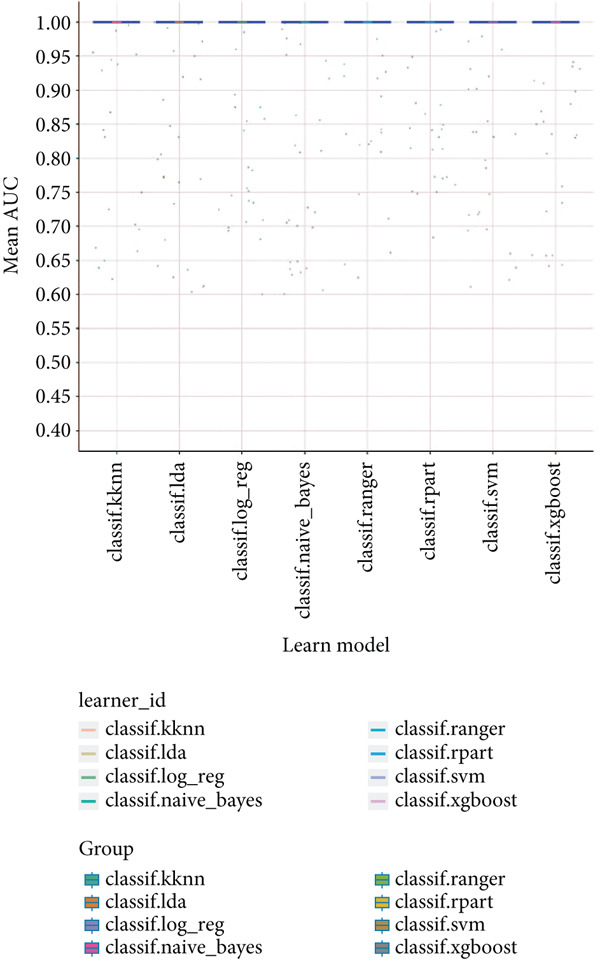
(a)

**Figure:**
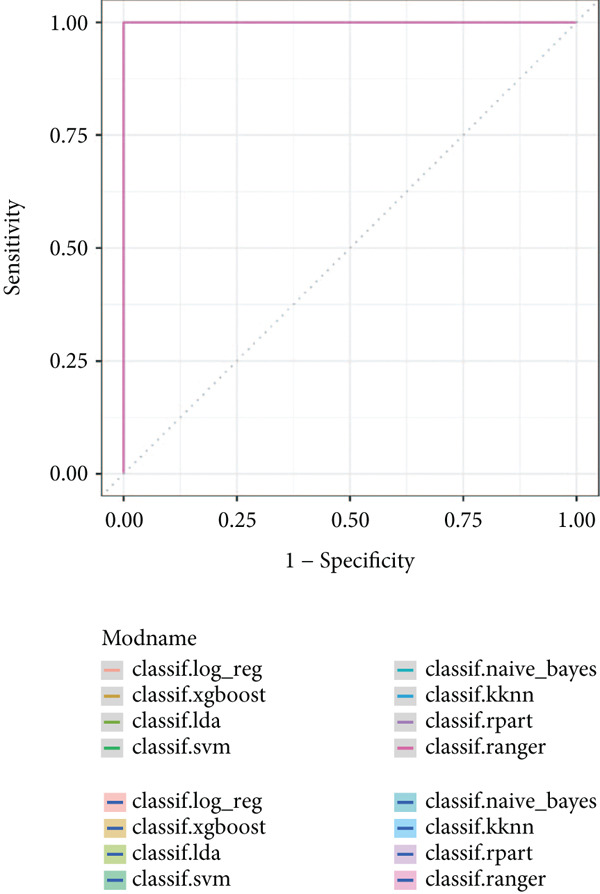
(b)

**Figure:**
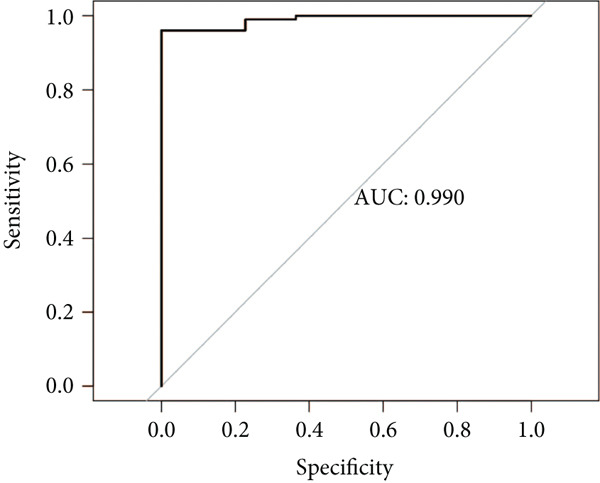
(c)

**Figure:**
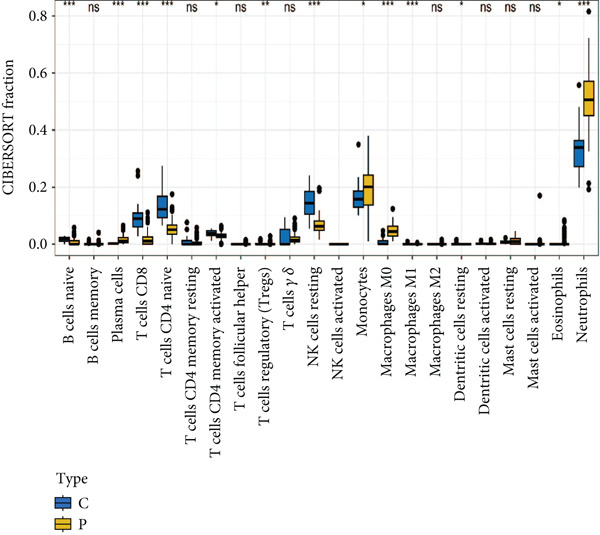
(d)

**Figure:**
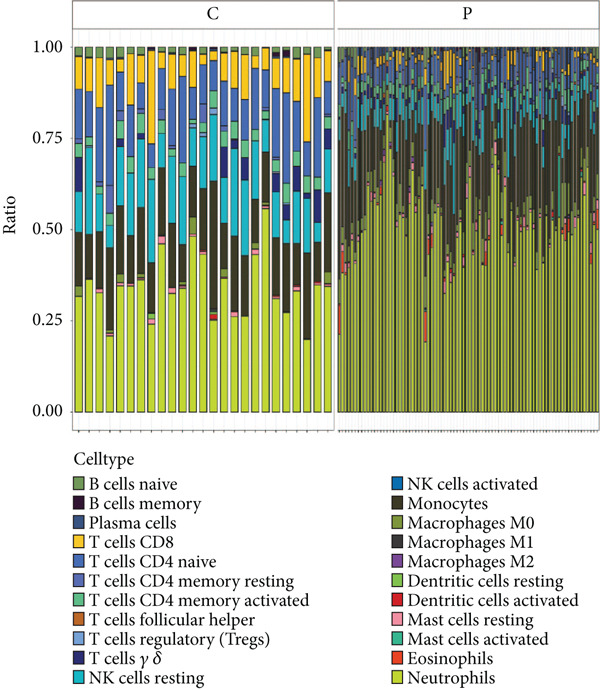
(e)

**Figure:**
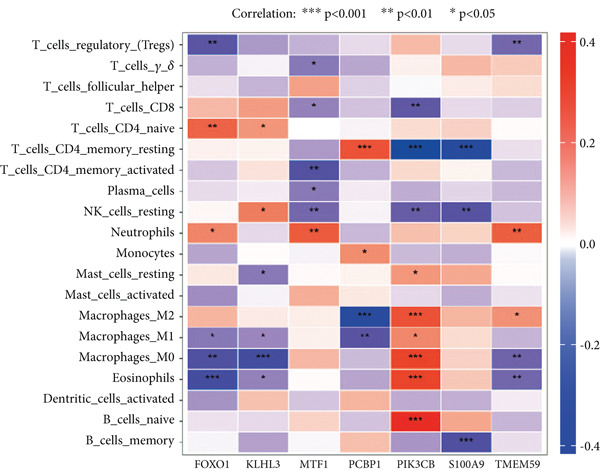
(f)

Figure 8Association between immune cells and seven feature genes. (a) The correlation coefficient of common immune cells with each feature gene and (b) the correlation heatmap of each feature gene and others.

**Figure:**
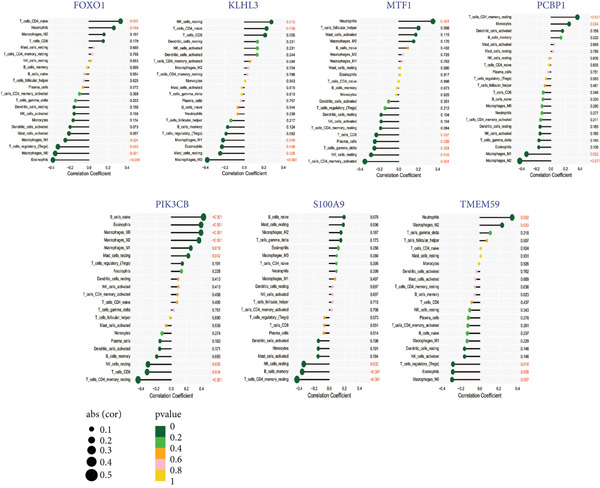
(a)

**Figure:**
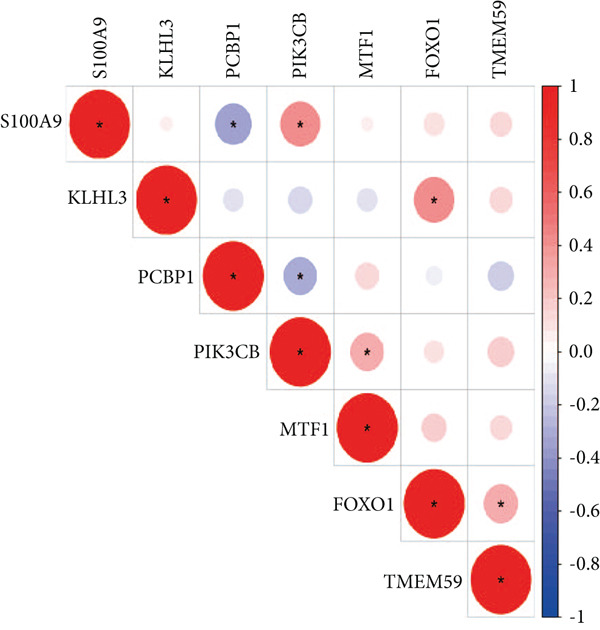
(b)

### 3.7. Identification of Hallmark Gene Sets in Sepsis

Hallmark gene sets comprise classical pathways integral to critical biological processes such as immune response, cell cycle regulation, oxidative phosphorylation, and mitochondrial metabolism, which are pivotal in influencing the progression and outcome of sepsis [21]. We further introduced ssGSEA to investigate the enrichment differences of these sets between the sepsis and control groups (Figure 9a). A large amount of hallmark gene sets displayed a significant difference between the sepsis and the control group, implying the functional association between these hallmark gene sets and sepsis progression. Additionally, the seven feature genes were related to several hallmark gene sets (Figure 9b). For instance, FOXO1 was inversely correlated with general hallmark gene sets such as UV response, reactive oxygen species pathway, mTorc1 signaling pathway, K‐RAS signaling pathway, HEME metabolism pathway, coagulation, cholesterol homeostasis, and adipogenesis. Notably, the seven feature genes showed a positive correlation with the TGF‐*β* signal pathway, which was downregulated in the sepsis group.

Figure 9Investigation of hallmark gene sets. (a) The distribution score of the hallmark gene sets in sepsis and control groups and (b) correlation heatmap of hallmark gene sets with seven feature genes.

**Figure:**
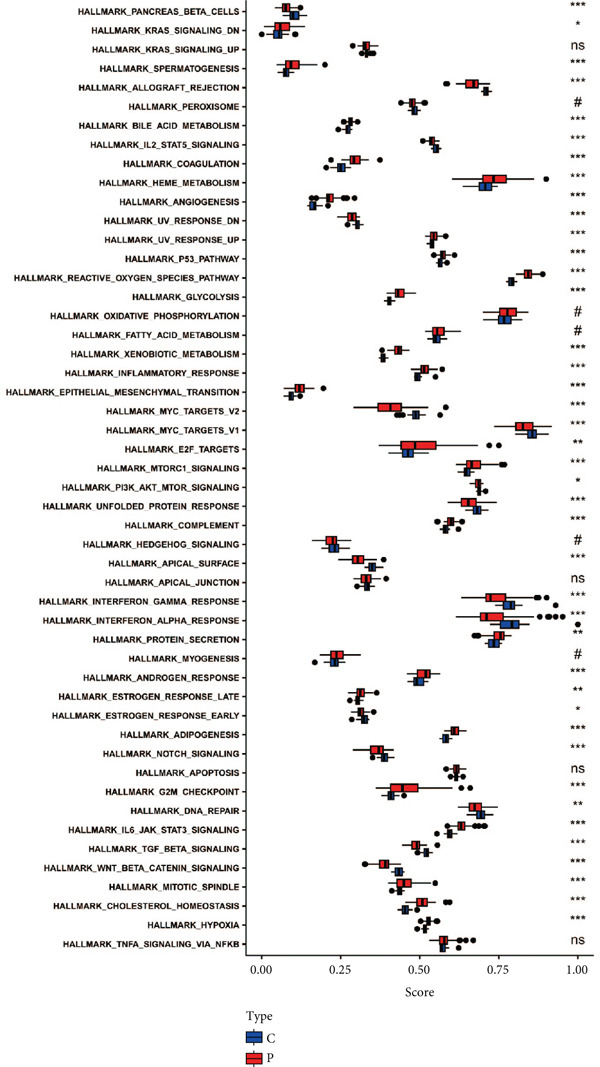
(a)

**Figure:**
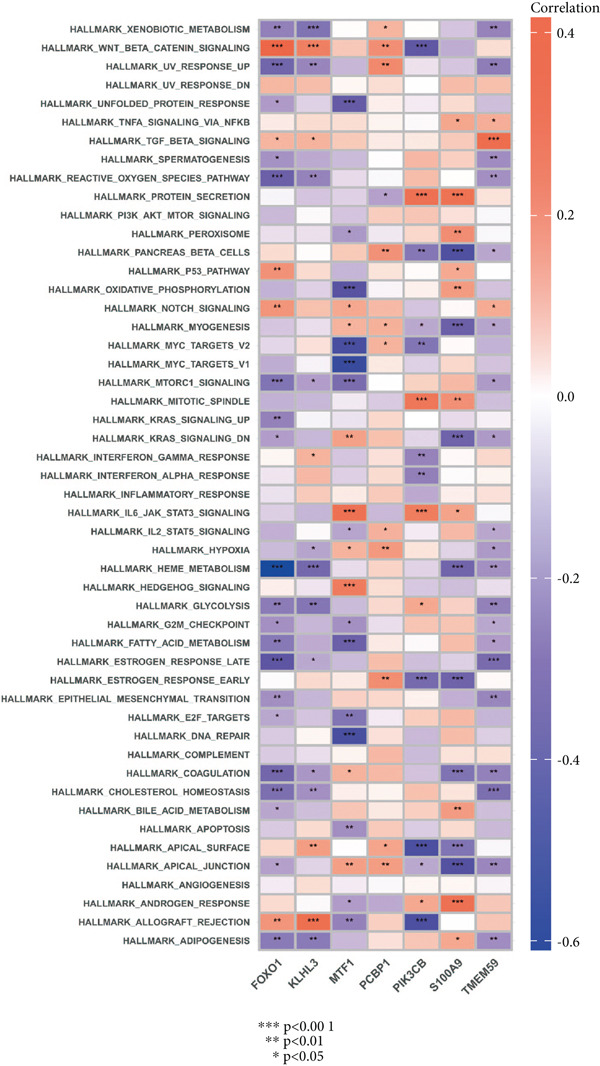
(b)

### 3.8. Function Validation of the Seven Feature Genes

**Figure 10:**
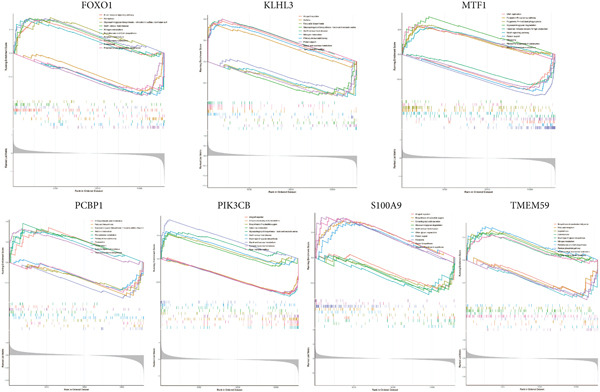
GSEA analysis of the seven genes.

Considering the high significance of the seven feature genes in assessing sepsis prognosis, we then conducted a GSEA survey to elucidate potential biological functions (Figure 10). Relying on median expression levels of the seven feature genes, sepsis samples were classified into two groups. B cell receptor signaling pathway, ferroptosis, glycosaminoglycan biosynthesis, graft‐versus‐host disease, nitrogen metabolism, pantothenate and CoA biosynthesis, porphyrin metabolism, primary immunodeficiency, proteasome, and proximal tubule bicarbonate reclamation were significantly different in the FOXO1 high group. Allograft rejection, asthma, fatty acid biosynthesis, glycosphingolipid biosynthesis, graft‐versus‐host disease, nitrogen metabolism, primary immunodeficiency, protein export, starch and sucrose metabolism, and Type I diabetes mellitus were associated with KLHL3 function. The predominant signaling pathways of other feature genes were also displayed (Figure 10). Detailed results of the GSEA analysis can be found in Table S4.

### 3.9. Seven Characteristic Genes Were Validated at the scRNA‐Seq Level and in Blood Samples

To validate the function of the seven feature genes, we explored the expression of the seven feature genes in the GSE167363 dataset from the scRNA‐seq dataset. First, the overall cell was clustered into six major cell populations including B cell, monocyte, neutrophil, NK cell, platelet, and T cell (Figure 11a). In the sepsis sample, neutrophils and platelets suggested a higher abundance (Figure 11b,c). Both S100A9 and KLHL3 had a strong association with neutrophils (Figure 11d). Moreover, we also surveyed the expression of the seven feature genes in six major cell types (Figure 11e,f). S100A9 had the highest transcript abundance, whereas KLHL3 had the lowest transcript abundance. PCBP11 and TMEM59 had almost equivalent distribution levels across the six cell types. qPCR results showed that the expression of S100A9, PCBP1, PIK3CB, FOXO1, and TMEM59 was consistent with the public dataset, among which S100A9, PIK3CB, and TMEM59 were significantly overexpressed in sepsis, whereas PCBP1 and FOXO1 were significantly underexpressed in sepsis (Figure S1).

Figure 11The expression of the seven feature genes in the scRNA‐seq dataset. (a) The cell cluster analysis, (b,c) the percentages of six types of cells in sepsis and control group, (d) the percentages of the seven feature genes across six types of cells, and (e,f) expression level of the seven feature genes across six types of cells.

**Figure:**
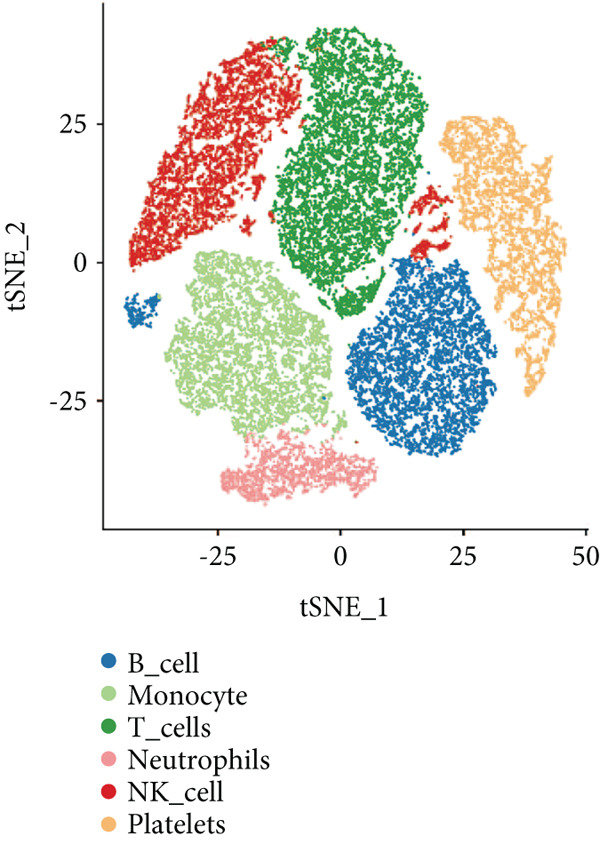
(a)

**Figure:**
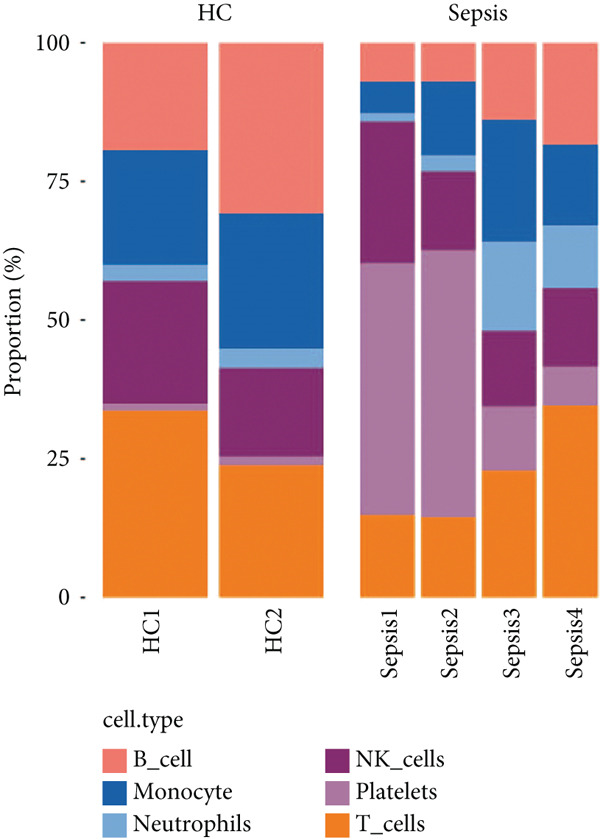
(b)

**Figure:**
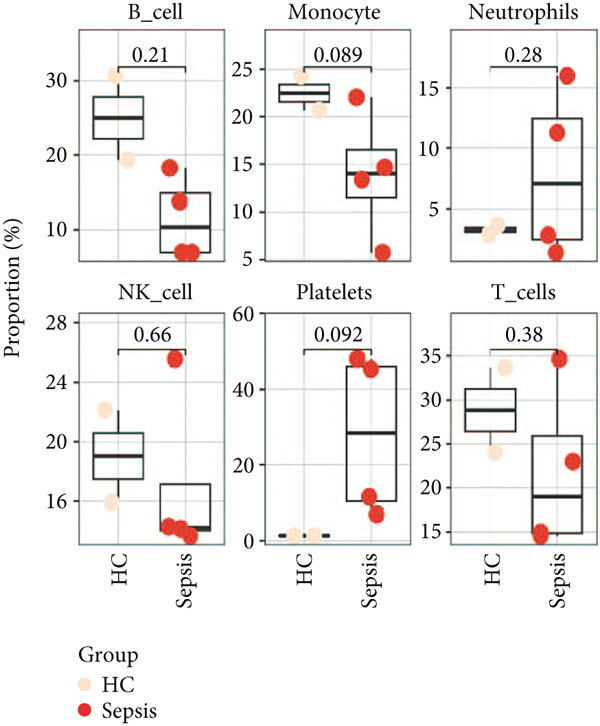
(c)

**Figure:**
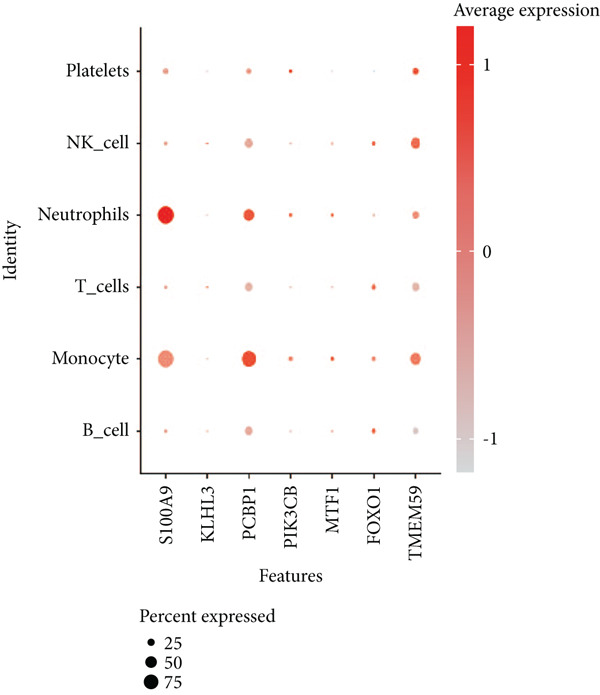
(d)

**Figure:**
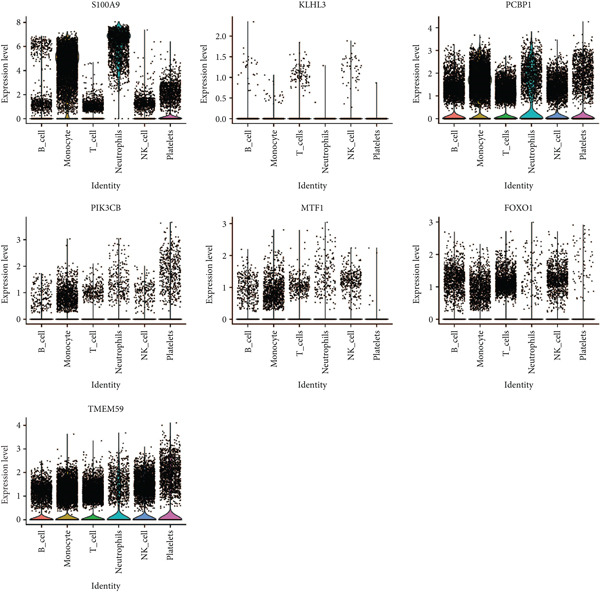
(e)

**Figure:**
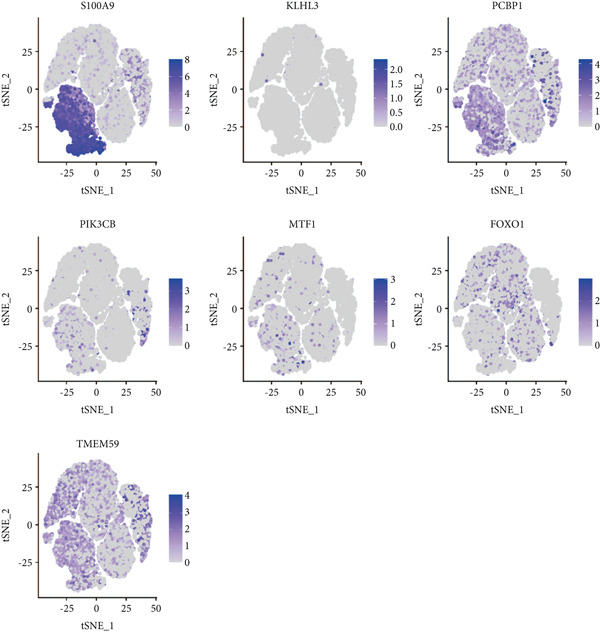
(f)

## 4. Discussion

Sepsis, a systemic inflammatory response to infection, remains a significant cause of morbidity and mortality worldwide. The intricate interplay of molecular mechanisms underlying sepsis has been a hot subject of extensive research, intended to improve diagnostic accuracy, prognostication, and therapeutic interventions [22]. Our study, focusing on the role of PCDGs in sepsis, provides a comprehensive insight into the molecular landscape of this complex condition.

Our initial foray into the differential expression analysis revealed a total of 3046 DEGs between sepsis and control groups. The upregulation of 1389 genes and the downregulation of 1657 genes in sepsis paints a picture of a cellular environment in [22] association with these DEGs with pathways such as fatty acid biosynthesis, graft‐versus‐host disease, and the PPAR signaling pathway is particularly intriguing. Fatty acid biosynthesis, for instance, has been implicated in the inflammatory response, and its dysregulation might contribute to the metabolic alterations observed in septic patients [23, 24]. Acute kidney injury (AKI) exacerbates the condition, leading to increased morbidity and mortality. Fatty acid–binding protein 4 (FABP4) levels were found to be predominantly elevated in renal tubular epithelial cells (RTECs) during septic AKI [25]. FABP4 exacerbates inflammation and apoptosis in the kidneys during sepsis, whereas the deletion of FABP4 in RTECs showed protective effects against kidney damage. This detrimental effect of FABP4 is linked to the TLR4/c‐Jun signaling pathway, through which FABP4 and c‐Jun form a positive feedback loop, amplifying inflammation and apoptosis in septic AKI [25]. The PPAR signaling pathway, on the other hand, plays a pivotal role in glucose and lipid metabolism and inflammation when recognized with natural or synthetic ligands, suggesting that its dysregulation could be central to the pathogenesis of sepsis [26].

The immune dysregulation observed in sepsis is well documented. Our findings, which highlight the upregulation of pathways related to antigen processing and presentation, autoimmune thyroid disease, and Th1, Th2, and Th17 cell differentiation, align seamlessly with this established narrative. This immune imbalance, characterized by both hyperinflammatory and immunosuppressive phases, is a hallmark of sepsis and contributes significantly to its morbidity and mortality [27, 28]. A hyperinflammatory environment leads to multiple organ damage and following nosocomial infection. Introducing pro‐ and anti‐inflammatory markers such as chemokines and cytokines as diagnostic biomarkers for diagnosing sepsis in clinical examination has been reported to decrease the organ dysfunction rate before lethal sepsis [27, 29]. Furthermore, the downregulation of metabolic pathways, such as fatty acid biosynthesis and the PPAR signaling pathway, resonates with previous studies that have emphasized the metabolic disturbances that accompany sepsis [30, 31]. This metabolic–immune interface, where pathways of energy production and utilization intersect with immune responses, is emerging as a critical area of sepsis research. To clarify the PCD molecular landscape patterns in sepsis, we identified 262 hub genes by taking the intersection between DEGs, PCDGs, and the genes of the turquoise module derived from WGCNA analysis, which were central within their respective modules. These hub genes were intricately associated with apoptotic signaling pathways, lysosomal membranes, and inflammatory diseases, which played a pivotal role in diverse PCD programs. For instance, in a cecal ligation and puncture (CLP) experimental sepsis animal model, inhibiting PDL1 relieved lung injury and restrained neutrophil lung infiltration, which led to low mortality [32]. PDL1 was found to be highly expressed in human neutrophils of septic patients and high PDL1 expression contributed to neutrophil survival by activating the PI3K‐dependent AKT phosphorylation [32].

Our study innovatively integrated multi‐omics transcriptome data and applied eight machine learning methods to pinpoint seven PRGs, including FOXO1, KLHL3, PCBP1, MTF1, TMEM59, PIK3CB, and S100A9. These feature genes were further validated via scRNA‐seq, confirming their cell‐type‐specific expression patterns and enhancing the reliability of our findings. The diagnostic prowess of these genes, evidenced by AUC values exceeding 0.9, highlights their potential as invaluable tools in clinical settings, aiding in the early identification of septic patients and guiding therapeutic interventions. Notably, S100A9 was reported to regulate the intestinal environment: Mice lacking S100A9 displayed reduced CX3CR1 protein levels, IL10 and TGF mRNA expression, and fewer T‐reg cells in intestinal tissues, accompanied by a higher risk of fatal neonatal sepsis. Administering S100 at birth reversed these effects, reducing sepsis‐related mortality and improving weight gain [33].

The immune response in sepsis is a complex dance involving various cellular players. Our findings, which highlight the significant augmentation of neutrophils in sepsis, are in line with the recognized role of these cells in the condition [34, 35]. A recent study showed that there were diverse dysfunction variations of neutrophils at terminal sepsis, including apoptosis suppression, reduced chemotaxis ability, and sweeping infiltration into the tissues. Infiltrated neutrophils could exert immunomodulatory influence on surrounding T cells [36, 37]. Similarly, the high PDL1 expression mediated by p38*α*‐MSK1/‐MK2 in neutrophils led to damaged T cell activation and enlarged T cell apoptosis [36]. TGF‐*β* released by mesenchymal stem cells (MSCs) could activate the AKT/FOXO1 pathway in macrophages and trigger nuclear translocation of FOXO1, which promoted the shift of macrophages towards the M2‐like phenotype [38]. The M2‐like polarization showed reduced levels of pro‐inflammatory cytokines in sepsis [38]. In addition, our study also delved into hallmark gene sets, providing a broader perspective on the cellular processes affected by sepsis. We have found that the TGF‐*β* signaling gene set was decreased and the inflammatory response gene set was upregulated in sepsis. These data offer potential therapeutic avenues for sepsis management.

The differential enrichment of these gene sets between sepsis and control groups offered a panoramic view of the cellular environment in sepsis. Metabolism‐associated gene sets were differentially expressed, implying the important role of abnormal metabolism in sepsis conditions. The associations between the seven feature genes and these hallmark gene sets further suggested potential roles for these genes in various cellular processes. For example, we identified that KLHL3 was mainly engaged in fatty acid biosynthesis, glycosphingolipid biosynthesis, and nitrogen metabolism in sepsis samples.

The validation of our findings at the scRNA‐seq level adds a layer of robustness to the clinical significance of the seven feature genes. Notably, the platelet was found to be higher in sepsis. Platelet hyperactivation and subsequent immune response are increasingly recognized as key features in severe sepsis [39, 40]. Inhibiting NOD‐like receptor protein 3 inflammasome (NLRP3) could impede platelet activation, which lessened pulmonary edema, glomerular injury, and renal injury [41, 42].

The strong association of genes like S100A9 and KLHL3 with neutrophils, pivotal players in sepsis, suggests potential avenues for therapeutic targeting. Moving forward, functional studies exploring the roles of these genes in sepsis pathogenesis will be invaluable. Additionally, clinical trials assessing the utility of these genes as diagnostic or prognostic markers, or even as therapeutic targets, could pave the way for improved management of septic patients.

## 5. Conclusion

In summary, our study offers a deep dive into the molecular intricacies of sepsis, with a special emphasis on PCDGs. The insights gleaned from this research not only enhance our understanding of sepsis but also point toward potential avenues for therapeutic intervention and prognostication. However, the sample size of this study has limitations and needs to be validated in the future; at the same time, more mechanistic studies are needed to explain the ideas.

## Ethics Statement

This study was approved by the Ethics Committee of Shanghai Pudong New Area People′s Hospital (No. 2025‐LW‐05), and informed consent of all patients was obtained.

## Disclosure

All the authors contributed substantially to the work presented in this article. The corresponding author had full access to all of the data and the final responsibility for the decision to submit this article for publication.

## Conflicts of Interest

The authors declare no conflicts of interest.

## Author Contributions

Shiqiang Min: conceptualization, data curation, formal analysis, methodology, validation, visualization, writing—original draft, and experimental verification. Tao Zhang: validation, visualization, and writing—original draft. Song Chen: supervision, funding acquisition, and project administration.

## Funding

This study is funded by the Project of Discipline Leader Training Program in Shanghai Pudong New Area, No. PWRd2024‐12.

## Supporting Information

Additional supporting information can be found online in the Supporting Information section.

## Supporting information


**Supporting Information 1** Figure S1: qPCR was used to verify the expression of the seven key genes.

**Supporting Information 2** Table S1: The PCD‐related genes.

**Supporting Information 3** Table S2: Primer sequences for the seven key genes.

**Supporting Information 4** Table S3: Detailed results of the GSEA analysis of DEGs.

**Supporting Information 5** Table S4: Detailed results of the GSEA analysis of the seven key genes.

## Data Availability

Partial transcriptome data are from Gene Expression Omnibus (https://www.ncbi.nlm.nih.gov/). Further inquiries can be directed to the corresponding author.
