# Supplementary figures and images for: Molecular Landscape and Predictive Significance of Programmed Cell Death‐Related Genes in Sepsis
Source: Hum Mutat. 2026 Jan 13;2026:5280021. doi: 10.1155/humu/5280021 (PMC12800578; doi:10.1155/humu/5280021)

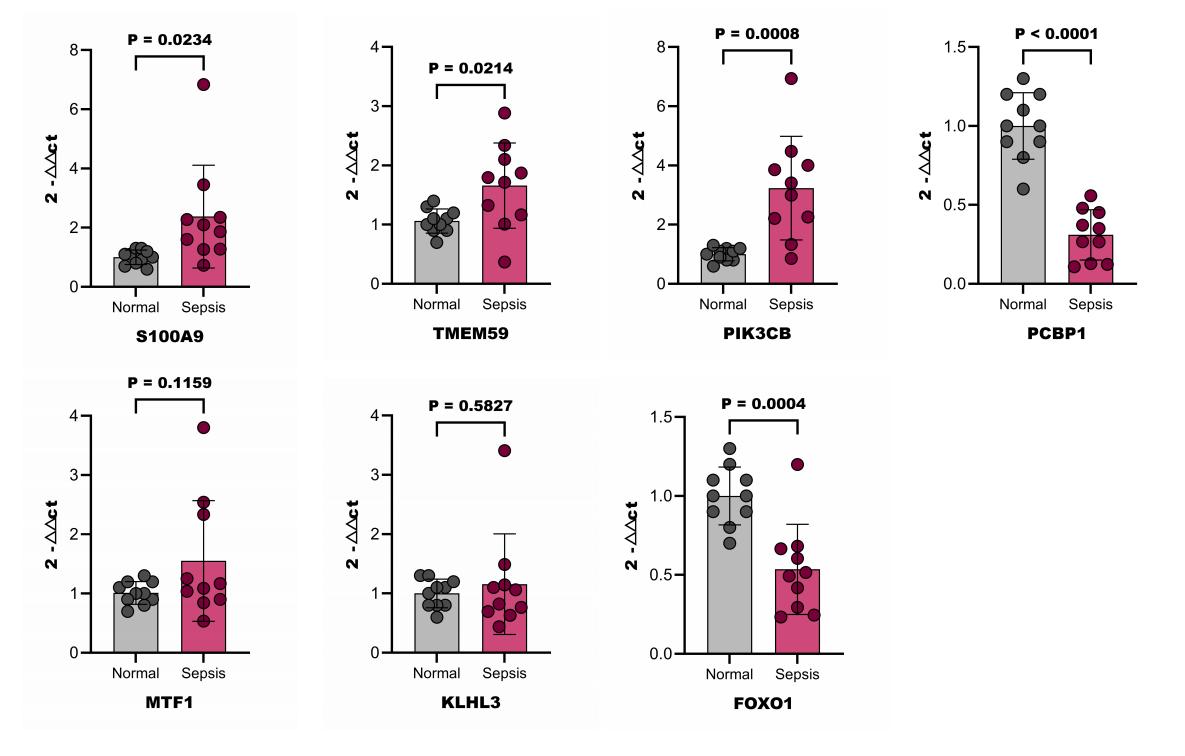

Supplement: Supplementary file 1 — Supporting Information 1 Figure S1: qPCR was used to verify the expression of the seven key genes. [file HUMU-2026-5280021-s004.jpg]
